# Effects of Short-Term Feeding of Resveratrol on Growth Performance, Meat Quality, Antioxidant Capacity, Serum Biochemical Parameters and Intestinal Health in Yellow-Feathered Broilers Under Dexamethasone-Induced Oxidative Stress

**DOI:** 10.3390/antiox14121459

**Published:** 2025-12-05

**Authors:** Hui Ye, Yangyu Wang, Huilan Zhu, Chao Huang, Weiwei Wang, Yifan Jia, Zhaoheng Hu, Huiyun Zhou, Shujie Liang, Chong Ling, Changming Zhang, Zemin Dong, Jianjun Zuo

**Affiliations:** 1 College of Animal Science, South China Agriculture University, Guangzhou 510642, China; magicsmall@scau.edu.cn (H.Y.); wangyangyu04@163.com (Y.W.); 20242025042@stu.scau.edu.cn (H.Z.); wangweiwei@scau.edu.cn (W.W.); 202119110606@stu.scau.edu.cn (Y.J.); huzhaoheng2024@163.com (Z.H.); lingchong@stu.scau.edu.cn (C.L.); zhangchangming@scau.edu.cn (C.Z.); zmdong@scau.edu.cn (Z.D.); 2 Graduate School, South China Agricultural University, Guangzhou 510642, China; huangchao@scau.edu.cn

**Keywords:** resveratrol, meatiness, oxidative stress, dexamethasone, antioxidant capacity

## Abstract

Oxidative stress is believed to deteriorate production performance and cause substantial economic losses in commercial poultry farming. Resveratrol (RES) is a polyphenolic antioxidant that can improve intestinal barrier function and regulate gut microbiota composition. This study aimed to evaluate whether short-term (14 days) dietary resveratrol (1000–3000 mg/kg) mitigates dexamethasone (DEX)-induced oxidative stress and performance loss in yellow-feathered broilers. Two hundred and forty 52-day-old birds were assigned to five treatments (*n* = 8 pens × 6). Control (CON) and DEX groups received the basal diet; DR1, DR2 and DR3 were provided with the basal diet plus 1000, 2000 or 3000 mg/kg RES. During days 1–5, the DEX and RES (DR1, DR2 and DR3) groups were intraperitoneally injected with 5 mg/kg BW DEX; CON birds received saline. DEX significantly reduced average daily gain (ADG) and raised feed conversion ratio (FCR) (*p* < 0.05) without altering feed intake. RES at 1000–2000 mg/kg improved ADG, reduced FCR and lowered serum corticosterone and blood urea nitrogen while increasing albumin (*p* < 0.05). DEX elevated malondialdehyde (MDA) in liver and thigh muscle, suppressed liver catalase (CAT) activity, and suppressed thigh muscle superoxide dismutase (SOD), glutathione peroxidase (GSH-Px) and CAT activities. In serum, only SOD activity decreased. RES partially alleviated the abnormal changes in these antioxidant indices. Intestinally, DEX increased MDA, shortened villi and reduced the villus-to-crypt ratio, whereas RES partially reinstated ileal morphology, decreased MDA dose-dependently and linearly enhanced duodenal SOD activity (*p* < 0.05). DEX downregulated Occludin mRNA; RES upregulated Occludin and elevated ileal GPX2, SOD, CAT and PPAR-γ transcripts with a quadratic response to RES dose, while lowering duodenal CAT mRNA. Overall, short-term RES supplementation—particularly at 1000–2000 mg/kg—improves growth performance, meat quality and intestinal health of yellow-feathered broilers under DEX-induced oxidative stress by enhancing systemic and intestinal antioxidant capacity and reinforcing epithelial barrier integrity.

## 1. Introduction

With population growth and improvements in living standards, the demand for animal products has continuously increased, leading to the rapid development of intensive farming practices over the recent few decades. The growth rate of livestock and poultry, feed conversion efficiency, and disease resistance have been significantly improved. However, under intensive production systems, poultry are more susceptible to various stressors during production and transportation, such as stocking density [1], thermal environment [2], and transport stress [3]. Under oxidative stress conditions, the excessive production of free radicals can lead to abnormalities in the digestive system and gut-derived infections [4], affect the absorption and utilization of nutrients [5], and reduce production performance [6]. Furthermore, prolonged oxidative stress can result in oxidative damage to animals, impaired immune function [7], and deteriorated meat quality [8]. Dexamethasone (DEX) is a synthetic glucocorticoid with potent immunosuppressive and anti-allergic effects [9]. It acts primarily by binding to the glucocorticoid receptor (GR) and regulating gene expression via genomic transactivation (GR homodimer binding to GREs) and transrepression of pro-inflammatory transcription factors such as NF-κB and AP-1. These actions suppress cytokine production and immune-cell activation, while shifting metabolism toward proteolysis and gluconeogenesis and potentially inducing oxidative stress [10,11,12]. It is commonly used as an immunosuppressive agent and as an inducer in oxidative stress models to study stress responses in poultry species simulating physiological and immune responses under oxidative stress conditions [13]. Previous studies have reported that oxidative stress can be induced by subcutaneous injection of DEX at 4.5 mg/kg once daily for 7 consecutive days in 21-week-old Hy-Line Brown laying hens [14], as well as by intramuscular injection of DEX (2 mg/kg BW) in 42-day-old male Arbor Acres broiler chickens to establish a chronic oxidative stress model [15], both achieving robust oxidative-stress induction. Considering that yellow-feathered broilers exhibit greater stress resistance [16], we therefore administered DEX at 5 mg/kg BW once daily for 5 consecutive days in the present study to ensure adequate model establishment. Identifying effective nutritional strategies to counteract oxidative stress, enhance antioxidant capacity, maintain immune function, and preserve gut health is crucial for improving poultry production efficiency and ensuring meat quality.

Resveratrol (RES, trans-3,4′,5-trihydroxystilbene) is a plant-derived, non-flavonoid stilbene polyphenol, found predominantly in human foods such as grape skins/seeds, Japanese knotweed rhizomes, peanuts, and certain berries [17]. As an antioxidant, RES can enhance the activity of endogenous antioxidant enzymes in chickens [18] and alleviate damage to intestinal morphology and barrier integrity in broilers under heat stress [19]. Under lipopolysaccharide (LPS) induction, RES has shown beneficial effects on the growth performance and intestinal health of yellow-feathered broilers [20]. Most previous poultry studies have emphasized lower-dose and longer-term RES supplementation, yet oral RES exhibits low bioavailability [21,22]; Therefore, higher dietary levels of RES are required to achieve pronounced beneficial effects, particularly under short-term feeding regimens [23]. In line with this, a previous study evaluating different dietary concentrations of resveratrol (0, 0.5, 1.0, 2.0, and 4.0 g/kg) in laying hens reported that 2.0 g/kg RES significantly reduced FCR [24]. To probe acute, dose-responsive protection under stress while minimizing confounding by long-term adaptation, we therefore adopted a short-term (14 d) regimen covering a higher dietary span (1000 mg/kg–3000 mg/kg). For the stressor, we used DEX 5 mg/kg once daily for 5 d, a schedule at the upper end of commonly reported poultry models [14,15,25], selected to produce a robust and reproducible oxidative-stress phenotype suitable for testing nutritional countermeasures without excessive mortality.

Therefore, this study aims to investigate the effects of short-term dietary RES supplementation on growth performance, meat quality, serum biochemical parameters, antioxidant capacity, and intestinal health in yellow-feathered broilers under DEX-induced oxidative stress. It also aims to determine whether short-term RES supplementation can mitigate the negative effects of oxidative stress and improve broiler health.

## 2. Materials and Methods

This experimental protocol was approved by the Ethics Committee and conducted under the supervision of the Animal Care and Use Committee at South China Agricultural University (Guangzhou, China, approval number: 2024f227. Date: 27 February 2024).

### 2.1. Experimental Design, Diet, and Management

A total of 240 healthy 45-day-old yellow-feathered broilers, evenly split between males and females, were randomly assigned to five treatment groups. Each treatment consisted of eight replicate pens with six birds per pen. The five treatment groups were designated as the Control group (CON), Dexamethasone group (DEX), DR1 group, DR2 group and DR3 group. The CON and DEX groups were fed a basal corn–soybean meal diet, while the DR1, DR2, and DR3 groups received the basal diet supplemented with 1000 mg/kg, 2000 mg/kg, and 3000 mg/kg RES, respectively. Following a 7-day acclimation period, the formal experiment commenced at 52 days of age and finished at 66 days of age. Starting on day 52, the CON group received intraperitoneal injections of normal saline (NS) at 5 mg/kg body weight per day for five consecutive days, whereas the other groups received DEX (5 mg/kg). The injections were discontinued on the sixth day. The rearing trials were conducted in the animal housing facilities of the College of Animal Science, South China Agricultural University. Throughout the study, broilers had ad libitum access to feed and water.

Resveratrol (purity ≥ 99%) was supplied by Guangzhou Jingjing Biotechnology Co., Ltd. (Guangzhou, China), is a white to light yellow powder, insoluble in water, easily soluble in ethanol, and odorless. The specific extraction process is as follows: using Giant Knotweed Rhizome as raw material, and after fermentation, extraction, concentration, dissolution, separation, filtration, concentration, separation, etc., resveratrol powder is finally obtained. DEX (2 mg/mL) was provided by Guangzhou Baiyunshan Tianxin Pharmaceutical Co., Ltd. (Guangzhou, China). The basal diet (Table 1) was formulated according to the nutritional guidelines recommended by the Ministry of Agriculture of the People’s Republic of China [26].

### 2.2. Sample Collection

Broilers from each group were weighed on days 1 (52 days old) and 15 (66 days old) of the experiment, and daily feed intake was recorded. On days 6 (57 days old) and 15, two birds with average body weights from each replicate were selected for sampling. Birds were feed-withdrawn for 12 h before weighing and sampling and were humanely euthanized by cervical dislocation. Ten milliliters of venous blood were collected from the neck vein of each selected bird and centrifuged at 3000 rpm for 10 min to obtain serum. Serum biochemical indicators related to oxidative reactions were measured. Subsequently, the birds were slaughtered, and the breast muscle, thigh muscle, liver, as well as segments of the duodenum, jejunum, and ileum were harvested. Mucosal samples from the duodenum, jejunum, and ileum were collected, and molecular samples of the duodenum and ileum were collected, with a portion stored at −80 °C for subsequent analysis of antioxidant capacity, meat quality, and intestinal morphology and expression of related genes. Individual-level measurements (e.g., serum biochemistry, qPCR, histology) were based on birds whose body weights were closest to the pen mean, with each sampled bird representing its corresponding pen. In these assays, the bird (selected in this way) was treated as the experimental unit.

### 2.3. Growth Performance Measurement

Before each weighing, or immediately upon any mortality, feed intake for each replicate and the body weight of the dead bird were recorded, and the number of live birds in that pen was updated in order to eliminate the influence of mortality on the calculations. Average daily gain (ADG), average daily feed intake (ADFI), and feed conversion ratio (FCR) were then calculated to evaluate growth performance. For growth-performance analysis, the pen was treated as the experimental unit. Feed intake and body-weight gain were expressed on a pen–day basis, using the actual number of live birds per pen per day as the denominator.

### 2.4. Antioxidant Capacity Assessment

Laboratory assays and histological measurements were performed using coded samples, and operators were blinded to treatment allocation during measurements.

Antioxidant status was evaluated by determining the activities of glutathione peroxidase (GSH-Px), superoxide dismutase (SOD), and catalase (CAT), and the concentration of malondialdehyde (MDA) in serum, leg muscle, and liver samples. In mucosal scrapings from the duodenum, jejunum, and ileum, SOD activity and MDA concentration were also measured. All assays were performed in accordance with the manufacturers’ instructions. MDA and CAT activity were measured using commercial kits from Beijing Solarbio Science & Technology Co., Ltd. (Beijing, China), while GSH-Px and SOD were determined with commercial kits from Nanjing Jiancheng Bioengineering Institute (Nanjing, China). Absorbance was read on a full-spectrum microplate reader (Multiskan™ GO, Thermo Fisher Scientific, Vantaa, Finland), and the measurement wavelengths for each assay followed the manufacturer’s instructions.

### 2.5. Serum Biochemical Parameters

Serum corticosterone (CS) was quantified using a commercial ELISA kit for chicken corticosterone (CSB-E11991C, Wuhan Huamei Biological, Wuhan, China). Serum albumin (ALB), globulin (GLB), glucose (Glu), and total protein (TP) concentrations were measured using commercial kits from Nanjing Jiancheng Bioengineering Institute (Nanjing, China). Creatinine (Cr) and blood urea nitrogen (BUN) were determined by colorimetric and urease methods, respectively, on an automated clinical chemistry analyzer (Cobas C311, Roche, Basel, Switzerland) at the Guangzhou Da’an Research Center (Guangzhou, China).

### 2.6. Meat Quality Evaluation

After sampling, breast and thigh muscles were allowed to bloom at room temperature for 45 min, and color parameters L* (lightness), a* (redness), and b* (yellowness) were measured using a colorimeter (OPTO-STAR, MATTHAUS, Eckelsheim, Germany). Additional portions were stored at 4 °C for 24 h and re-measured. Each sample was measured in triplicate. pH of the breast and thigh muscles was determined at 45 min postmortem and after 24 h at 4 °C using a pH meter (SevenCompact S220, Mettler Toledo, Greifensee, Switzerland) fitted with a glass spear-type penetration electrode. Trimmed muscle samples were weighed (W_0_) and stored at 4 °C for 24 h. Surface moisture was removed using filter paper, and the samples were weighed again (W_1_). Water loss percentage was calculated as follows: Water Loss (%) = [(W_0_ − W_1_)/W_0_] × 100% [27]. Shear force was measured using a muscle tenderness meter (Harbin Xiangfang Jinqiao Machinery Processing Factory, Harbin, China).

### 2.7. Intestinal Morphology Analysis

Sections approximately 2 cm in length from the mid-duodenum, proximal jejunum (at the 1/4 point), and mid-ileum were fixed in 4% paraformaldehyde-phosphate-buffered saline (pH 7.4). The fixed tissues were sent to Beijing Jiputeng Biotechnology Co., Ltd. (Beijing, China) for the preparation of 5 μm thick histological sections. Sections were examined on a Zeiss Axioskop 40FL fluorescence microscope (Jena, Germany) equipped with a Moticam 1080 digital camera (Richmond, BC, Canada) using a 50× objective. Image analysis was performed with Motic Advanced 3.0; prior to measurements, pixel-to-micrometer calibration was conducted in the software using a stage micrometer (0.01 mm/division). For each bird, as many well-oriented villi as possible and 40 well-oriented crypts were selected. Villus height and crypt depth were measured, and the villus-to-crypt ratio (V/C) was calculated.

### 2.8. Quantitative Real-Time PCR Analysis

Total RNA was extracted using the FastPure Total RNA Isolation Kit for Tissue (Vazyme, Nanjing, China). RNA purity and concentration were assessed with a micro-volume UV spectrophotometer (NanoDrop 2000, Thermo Scientific NanoDrop Products, Wilmington, DE, USA), accepting A260/280 = 1.80–2.20 and A260/230 ≥ 1.8. RNA integrity was examined by agarose gel electrophoresis; samples not meeting these criteria were re-extracted. cDNAs were synthesized from the extracted RNA using the EZscript Reverse Transcription Mix II (EZBioscience, Suzhou, China). Quantitative real-time PCR (qRT-PCR) was performed on a CFX96 Touch Real-Time PCR Detection System (Bio-Rad, Hercules, CA, USA) using the 2× Polarsignal SYBR Green qPCR Mix (MIKX, MKG800, Shenzhen, China). The primer sequences used for real-time PCR are listed in Table 2. Primer specificity and amplification efficiency were first evaluated using pooled cDNA samples. Primer pairs showing amplification efficiencies between 90% and 110% were considered acceptable and were retained for subsequent analyses. All qPCR reactions were performed in duplicate, and data were accepted only when the difference between technical replicates was ≤5%. A no-reverse transcription control, prepared from pooled RNA, was included to monitor genomic DNA contamination, and nuclease-free water was used as the no-template control. The thermal cycling protocol consisted of an initial denaturation at 95 °C for 1 min, followed by 40 cycles of 95 °C for 15 s, 55–62 °C for 30 s, and 72 °C for 45 s, and a final stage at 95 °C for 30 s, 60 °C for 30 s, and 95 °C for 30 s. Relative mRNA abundance was calculated using the 2^^−ΔΔCt^ method, with GAPDH serving as the housekeeping reference gene.

### 2.9. Statistical Analysis

Data were analyzed using GraphPad Prism 9.0 (GraphPad Software, La Jolla, CA, USA) for one-way analysis of variance (ANOVA) and for graph preparation. For the parameters measured in yellow-feathered broilers at 57 and 66 days of age, only one-way ANOVA was used for statistical analysis in the present study. Tukey’s multiple comparison test was applied to detect pair-wise differences among treatments. In addition, orthogonal polynomial trend analyses (linear and quadratic) were carried out in IBM SPSS Statistics 27.0 to evaluate dose–response patterns. Differences were considered significant at *p* < 0.05, and a tendency was noted for 0.05 ≤ *p* < 0.10. All results are expressed as the mean ± standard error of the mean (SEM).

## 3. Results

### 3.1. Growth Performance

The analysis of growth performance (Table 3) revealed that DEX significantly decreased the ADG and reduced body weight on day 14 of the trial of yellow-feathered broilers from 52 to 66 days of age (*p* < 0.05) without having a significant effect on the ADFI during the same period. Additionally, DEX treatment significantly increased the FCR from 52 to 66 days of age (*p* < 0.05). During the injection phase (days 1–5), DEX markedly impaired body-weight accretion, with some birds exhibiting net weight loss. Dietary supplementation with 1000 and 2000 mg/kg RES notably alleviated the negative impact of DEX on ADG between 52 and 66 days of age (*p* < 0.05). Furthermore, the addition of 1000 and 2000 mg/kg RES significantly mitigated the DEX-induced increase in FCR (*p* < 0.05). Orthogonal polynomial contrast revealed a significant quadratic dose-response for both ADG and FCR (*p* < 0.01), indicating that the improvements peaked at 1000 and 2000 mg/kg RES.

### 3.2. Serum Biochemical Parameters

Analysis of serum biochemical parameters (Table 4) revealed that in 57-day-old yellow-feathered broilers, DEX stimulation significantly increased serum CS levels (*p* < 0.05). Concurrently, dietary supplementation with 1000, 2000, and 3000 mg/kg RES significantly mitigated the DEX-induced elevation in CS concentrations (*p* < 0.05). CS exhibited a significant linear decrease accompanied by a quadratic effect (linear and quadratic *p* < 0.01). At 66 days of age, DEX stimulation significantly raised serum CS and BUN levels (*p* < 0.05). During the same period, supplementation with 1000 mg/kg RES significantly increased serum ALB levels while reducing CS and BUN (*p* < 0.05); supplementation with 2000 mg/kg RES significantly elevated both serum ALB and TP levels and reduced BUN (*p* < 0.05); and supplementation with 3000 mg/kg RES significantly increased serum TP levels while lowering CS and BUN (*p* < 0.05). TP was linearly increased with RES supplementation (*p* < 0.01). With increasing RES dosage, CS showed a significant linear decline (*p* < 0.01), and BUN exhibited a significant linear decline accompanied by a quadratic response (linear & quadratic *p* < 0.01). ALB displayed a significant quadratic response (*p* < 0.01), peaking at 1000 and 2000 mg/kg.

### 3.3. Antioxidant Capacity

Assessment of serum and tissue antioxidant capacity (Table 5) indicated that DEX stimulation significantly increased MDA concentrations in the liver and thigh muscle of 66-day-old yellow-feathered broilers (*p* < 0.05), while significantly reducing SOD activity in both the thigh muscle and serum, as well as decreasing CAT and GSH-Px activities in the thigh muscle (*p* < 0.05). Dietary supplementation with 1000 mg/kg RES significantly enhanced serum GSH-Px, liver SOD and GSH-Px, and thigh muscle SOD, CAT, and GSH-Px activities compared to DEX group (*p* < 0.05). In birds receiving 2000 mg/kg RES, serum SOD, liver SOD, and thigh muscle SOD, CAT, and GSH-Px activities were significantly increased compared to DEX group (*p* < 0.05), with a trend toward reducing thigh muscle MDA levels (*p* = 0.052) and enhancing serum GSH-Px activity (*p* = 0.081). Moreover, dietary inclusion of 3000 mg/kg RES significantly reduced thigh muscle MDA levels compared to DEX group (*p* < 0.05) and markedly increased liver SOD, as well as thigh muscle SOD and GSH-Px activities (*p* < 0.05), with a trend toward elevating serum (*p* = 0.079) and liver (*p* = 0.081) GSH-Px activities and thigh muscle CAT activity (*p* = 0.063). Serum SOD activity increased linearly with dietary RES supplementation (*p* = 0.01) and displayed an additional quadratic component (*p* = 0.01). Serum GSH-Px activity followed a pronounced quadratic curve (*p* < 0.01). Liver SOD activity rose linearly with dose and also showed a quadratic response (linear and quadratic *p* < 0.01), whereas hepatic GSH-Px exhibited a significant quadratic effect (*p* = 0.03). In the thigh muscle, MDA concentration declined linearly as RES increased (*p* < 0.01), while SOD, CAT, and GSH-Px activities all increased linearly and were accompanied by quadratic effects (linear and quadratic *p* < 0.01).

Analysis of intestinal antioxidant capacity (Table 6) revealed that DEX administration significantly increased MDA concentrations in the duodenum, jejunum, and ileum of 66-day-old yellow-feathered broilers (*p* < 0.05). Dietary supplementation with 1000 mg/kg RES significantly reduced MDA levels in the jejunum and ileum under DEX stimulation (*p* < 0.05), with a trend toward decreasing duodenal MDA levels (*p* = 0.083). In birds fed 2000 mg/kg RES, jejunal MDA levels were significantly decreased under DEX stimulation (*p* < 0.05), with trends toward reducing duodenal MDA levels (*p* = 0.097) and increasing duodenal SOD activity (*p* = 0.082). Furthermore, dietary inclusion of 3000 mg/kg RES significantly reduced jejunal MDA levels and significantly increased duodenal SOD activity under DEX stimulation (*p* < 0.05). For intestinal antioxidant parameters, only a few variables displayed significant dose-related trends. Duodenal SOD activity increased linearly with RES supplementation (*p* = 0.01). Jejunal MDA concentration declined linearly and showed a significant quadratic response (linear and quadratic *p* < 0.01).

### 3.4. Meat Quality

Meat quality analysis (Table 7) showed that in 66-day-old yellow-feathered broilers, DEX challenge significantly reduced the a* and b* values of the thigh muscle and tended to decrease the L* value, while significantly increasing the pH values at 45 min and 24 h post-slaughter (*p* < 0.05). In addition, the a*, L*, and b* values of the pectoralis were significantly decreased (*p* < 0.05).

Dietary supplementation with 1000 mg/kg RES significantly alleviated the abnormal changes induced by DEX in the b* value and pH_45min_ of the thigh muscle as well as in the a*, L*, and pH_45min_ of the pectoralis. It also significantly reduced the drip loss of the thigh muscle compared to DEX group (*p* < 0.05) and tended to mitigate the abnormal changes in the thigh muscle a* (*p* = 0.096) and L* (*p* = 0.094) values. Supplementation with 2000 mg/kg RES significantly ameliorated the abnormal changes in the L* value and pH_45min_ of the thigh muscle and in the L* value of the pectoralis, significantly reduced thigh muscle drip loss (*p* < 0.05), and tended to alleviate the abnormal changes in the thigh muscle a* (*p* = 0.057) and the pectoralis pH_45min_ (*p* = 0.058). Moreover, dietary supplementation with 3000 mg/kg RES significantly mitigated the abnormal changes in the a*, b*, and pH_45min_ of the thigh muscle as well as in the L*, b*, and pH_45min_ of the pectoralis, significantly reduced thigh muscle drip loss (*p* < 0.05), and tended to reduce the shear force of the pectoralis (*p* = 0.091).

In the thigh muscle, the a* and b* values increased linearly with dietary RES supplementation (*p* < 0.01), whereas the L* value displayed a significant quadratic response (*p* < 0.01). The pH_45min_ declined linearly as the RES dose increased (*p* < 0.01) and also showed a quadratic component (*p* = 0.02). Drip loss percentage decreased linearly with rising RES levels (*p* < 0.01). In the pectoralis, both the b* and L* values improved linearly with increasing RES (*p* < 0.01); the L* value additionally exhibited a significant quadratic response (*p* = 0.01). The pH_45min_ fell linearly as the RES dose increased (*p* < 0.01).

### 3.5. Intestinal Morphology

**Figure 1:**
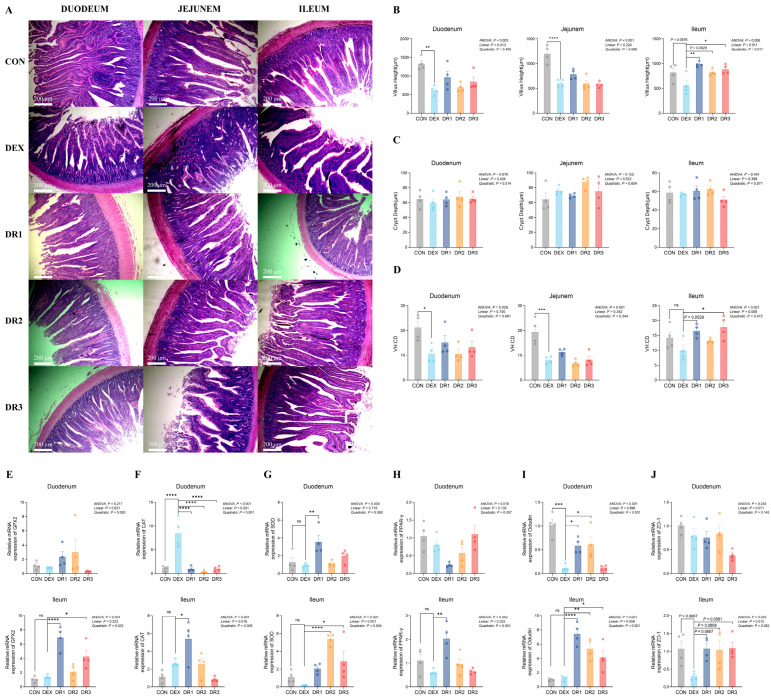
Short-term feeding of resveratrol significantly improves intestinal morphology, and related gene expression in dexamethasone-induced yellow-feathered broilers. (**A**) Cross-sectional images of the duodenum, jejunum, and ileum of broilers in different treatment groups (magnification 50×; scale bar = 200 μm). (**B**) Villus height (*n* = 4). (**C**) Crypt depth (*n* = 4). (**D**) VH: CD (*n* = 4). (**E**) Relative mRNA expression of GPX2 (*n* = 4). (**F**) Relative mRNA expression of CAT (*n* = 4). (**G**) Relative mRNA expression of SOD (*n* = 4). (**H**) Relative mRNA expression of PPAR-γ (*n* = 4). (**I**) Relative mRNA expression of Occludin (*n* = 4). (**J**) Relative mRNA expression of ZO-1 (*n* = 4). The data are shown as the means  ±  SEM. * *p* < 0.05, ** *p* < 0.01, *** *p* < 0.001, **** *p* < 0.0001. ANOVA, *p* value for the overall treatment effect from a one-way analysis of variance; linear, *p* value for the linear orthogonal polynomial contrast across dietary RES doses; quadratic, *p* value for the quadratic orthogonal polynomial contrast across dietary RES doses.

Intestinal morphology analysis (Figure 1A–D) revealed that DEX stimulation significantly reduced villus height in the duodenum and jejunum of 67-day-old broilers (*p* < 0.05) and showed a trend towards reducing villus height in the ileum (*p* = 0.098). Additionally, DEX significantly decreased the ratio of villus height to crypt depth in the duodenum and jejunum (*p* < 0.05).

Dietary supplementation with 1000 mg/kg and 3000 mg/kg RES significantly mitigated the DEX-induced reduction in villus height in the ileum (*p* < 0.05), while 2000 mg/kg RES showed a trend towards alleviating this effect (*p* = 0.093). Furthermore, supplementation with 3000 mg/kg RES significantly increased the ratio of villus height to crypt depth in the ileum under DEX stimulation (*p* < 0.05), and 1000 mg/kg RES showed a trend towards increasing this ratio (*p* = 0.053). In the ileum, villus height increased linearly with increasing dietary RES dose and was accompanied by a significant quadratic component (linear and quadratic *p* = 0.01).

### 3.6. Relative mRNA Expression

Analysis of intestinal gene mRNA expression (Figure 1E–J) revealed that DEX stimulation significantly decreased Occludin mRNA expression in the duodenum of 66-day-old broilers (*p* < 0.05), while increasing CAT mRNA expression in the duodenum and showing a trend toward lower ZO-1 mRNA expression in the ileum (*p* = 0.066).

Compared with the DEX group, 1000 mg/kg RES supplementation significantly increased SOD and Occludin mRNA expression in the duodenum, as well as GPX2, CAT, PPAR-γ, and Occludin mRNA expression in the ileum (*p* < 0.05). It also lowered CAT mRNA expression in the duodenum and showed a trend toward higher ZO-1 mRNA expression in the ileum (*p* = 0.066). Similarly, 2000 mg/kg RES supplementation markedly elevated Occludin mRNA expression in the duodenum and SOD and Occludin mRNA expression in the ileum (*p* < 0.05). In addition, it reduced CAT mRNA expression in the duodenum (*p* < 0.05) and showed a trend toward increased ZO-1 mRNA expression in the ileum (*p* = 0.081). Finally, 3000 mg/kg RES supplementation significantly increased GPX2, SOD, and Occludin mRNA expression in the ileum (*p* < 0.05), lowered CAT mRNA expression in the duodenum (*p* < 0.05), and showed a trend toward higher ZO-1 mRNA expression in the ileum (*p* = 0.058). Ileal GPX2 mRNA displayed a significant quadratic response (*p* = 0.02). Jejunal CAT mRNA showed both linear and quadratic trends (linear and quadratic *p* < 0.01), whereas ileal CAT mRNA exhibited a significant linear effect (*p* = 0.02) together with a quadratic component (*p* < 0.01). Ileal SOD mRNA likewise demonstrated significant linear and quadratic effects (both *p* < 0.01). Finally, Occludin mRNA in both the jejunum and ileum followed a pronounced quadratic pattern (*p* < 0.01).

## 4. Discussion

This study aimed to investigate the adverse effects of a DEX-induced oxidative stress model in yellow-feathered broilers, including its impact on growth performance, meat quality, serum biochemical parameters, antioxidant capacity, and intestinal health. It also examined whether short-term RES supplementation could alleviate oxidative stress in these broilers. The results showed that DEX-induced oxidative stress inhibited growth and development, compromised meat quality and antioxidant performance, and damaged intestinal morphology by impairing antioxidant capacity and barrier function in the intestine. In contrast, short-term RES supplementation significantly improved daily weight gain, pectoralis and thigh muscle quality, antioxidant capacity, and intestinal health in broilers under oxidative stress. This study demonstrates for the first time that short-term (14 days) feeding of resveratrol can significantly improve the production performance and meat quality of broilers under oxidative stress during the pre-marketing stage. These findings provide new insights into short-term strategies for mitigating oxidative stress in broilers, especially regarding growth performance and meat quality—areas of key interest to the poultry industry.

In poultry production, factors such as transportation, high temperatures, and bacterial infections often induce oxidative stress, negatively affecting both meat quality and body weight, particularly near market age, thus reducing economic returns [28]. In this study, we used DEX to establish an oxidative stress model, following approaches similar to other research that injected DEX to explore physiological responses under oxidative stress [13]. As expected, broilers at 57 and 66 days of age exhibited significantly elevated serum CS levels under DEX induction, a commonly used indicator of oxidative stress [29]. This confirmed that DEX injection successfully induced oxidative stress in our experimental broilers.

Our results demonstrated that in yellow-feathered broilers under DEX-induced oxidative stress, ADG declined markedly while FCR rose significantly, with no evident changes in ADFI. Especially during the period of DEX administration, broilers in the DEX group exhibited a pronounced decline in body weight, with ADG even becoming negative, whereas ADFI was slightly higher than that of the control group. This pattern suggests that under intense DEX stimulation, nutrient absorption in broilers was impaired, leading to a deterioration in growth performance. These findings align with some previous studies suggesting that DEX may inhibit growth by interfering with nutrient absorption, rather than reducing feed intake [30,31]—an effect supported by research showing that DEX downregulates nutrient transporter genes [5]. However, some studies have also observed significantly reduced ADFI under DEX stimulation [32], which may relate to dosage. For instance, injecting 1 mg/kg DEX significantly lowered both ADG and ADFI [32], whereas 3 mg/kg DEX reduced only ADG [31]. In our study, 5 mg/kg of DEX impaired nutrient absorption and utilization without affecting feed intake. Nevertheless, diets supplemented with 1000 mg/kg or 2000 mg/kg RES significantly improved ADG and FCR under DEX-induced stress, indicating that RES can partially mitigate oxidative stress-related declines in growth performance. This aligns with prior reports showing RES improves growth performance under challenges such as LPS [33], *Escherichia coli* infection [34], and heat stress [35], and it is particularly notable that our feeding period was even shorter—an important factor for enhancing economic efficiency.

As living standards rise, consumer demand for high-quality meat continues to grow, yet oxidative stress from factors such as heat, mycotoxins, and transportation can compromise meat quality at market age [8]. Our findings revealed that under DEX-induced oxidative stress, the a*, b*, and pH (both 45 min and 24 h) values of the thigh muscle were significantly affected, as were the a*, b*, and L* values in the breast muscle. Meat color, tenderness, and water-holding capacity are crucial determinants of meat quality [36]. With RES supplementation, thigh muscle a*, L*, b*, pH45min, and drip loss were notably improved, and pectoralis a*, L*, b*, and pH45min also showed significant improvement compared to DEX-stressed broilers. Specifically, 1000 mg/kg RES proved most effective for improving thigh muscle b* and pectoralis a*, 2000 mg/kg RES worked best for thigh muscle L*, and 3000 mg/kg RES excelled in enhancing thigh muscle a*, b* and pectoralis b*. Improvements in meat color likely stem from heightened antioxidant enzyme activity that reduces oxidative damage in muscle tissue. Consistent with our study, previous research reported that RES ameliorates meat color, pH, and drip loss in broilers while improving overall antioxidant capacity [37]. Other studies have shown that a 49-day RES treatment improves pork quality [38], and 21 days of RES supplementation benefits meat quality in heat-stressed broilers [39]; our study indicates that even a shorter 14-day treatment can improve meat quality.

Such improvements in meat quality likely arise from greater antioxidant capacity conferred by RES. External stressors can lead to excessive reactive oxygen species (ROS), which damage cellular membranes and tissues, disrupt physiological functions, and undermine the antioxidant system. Typically, ROS are first converted into hydrogen peroxide via SOD and then further reduced to water and oxygen by peroxidases, CAT, and GSH-Px [40]. MDA, a biomarker of lipid peroxidation, reflects the extent of oxidative stress [41]. In our study, we measured MDA, SOD, GSH-Px, and CAT in the serum, liver, and thigh muscle. Under DEX induction, serum SOD levels declined significantly; liver MDA rose considerably; and thigh muscle MDA increased markedly while SOD, GSH-Px, and CAT decreased. These changes indicate that DEX triggers oxidative stress, damaging the antioxidant capacity of serum, liver, and thigh muscle, consistent with prior findings [42]. Reduced antioxidant capacity in muscle can impair meat quality, as observed in our results [43]. Insufficient antioxidant defenses allow ROS to accumulate, diminishing growth performance and deteriorating meat quality—particularly in the thigh muscle [43]. However, RES supplementation notably alleviated the decline in serum SOD, the increase in thigh muscle MDA, and reductions in thigh muscle SOD, GSH-Px, and CAT. It also elevated serum GSH-Px as well as liver SOD and GSH-Px, underscoring the effectiveness of RES in mitigating oxidative stress and preserving antioxidant function in broilers. Other research has shown that RES enhances total antioxidant capacity (T-AOC) and CAT activity while reducing MDA levels in broiler breast muscle [37], and that it counters the drop in T-AOC, CAT, and GSH-Px activities under heat stress [39]. Our study confirms that even short-term RES supplementation can partially counteract damage to antioxidant capacity, thereby contributing significantly to economic returns.

Serum biochemical parameters are key indicators of an animal’s health status. Total serum protein consists of ALB and GLB; ALB levels correlate with liver and kidney function and nutritional status, while GLB serves as a precursor to immunoglobulins (e.g., IgG), reflecting immune status and physiological condition [44]. BUN can indirectly signal kidney function, and abnormal values often suggest renal impairment [45]. In our study, DEX markedly increased serum BUN, revealing potential kidney damage under oxidative stress. RES supplementation substantially decreased BUN levels and raised ALB levels, indicating improved liver and kidney function and reduced oxidative damage to the liver. These outcomes agree with our earlier findings on liver antioxidant capacity. Previous research also shows that RES effectively alleviates liver immune damage and inflammation, as well as oxidative stress in poultry organs [46].

However, in the physiological context of birds as uricotelic species, uric acid (UA) is the principal nitrogenous end-product and the most specific routine marker of renal excretory function, whereas BUN and creatinine are less specific indicators in avian species [47]. Nonetheless, under DEX challenge or other catabolic stressors, increases in BUN and creatinine have been reported in broilers, indicating enhanced proteolysis and altered renal load/filtration; when interpreted together with endocrine and oxidative stress markers, BUN still provides useful information as a stress-responsive indicator [48,49]. A limitation of the present study is that UA was not measured; future work should include UA to strengthen the interpretation of renal function in the avian context.

The intestine is a key organ for digestion and absorption and thus plays a fundamental role in animal nutrition and overall health. Our previous work indicated that although DEX treatment did not significantly alter feed intake, it markedly suppressed weight gain, potentially due to impaired nutrient absorption in the small intestine [50]. Indeed, the oxidative state and structural integrity of the small intestine are crucial for normal digestive and absorptive functions [51]. In this study, DEX significantly elevated MDA levels in the duodenum, jejunum, and ileum, exacerbating oxidative stress; RES supplementation alleviated these increases and enhanced duodenal SOD levels, possibly supporting nutrient digestion and absorption. These results align with earlier findings where RES supplementation alleviated aberrant changes in SOD and CAT activity and MDA levels in the jejunum of heat-stressed ducks [52]. Villus height, crypt depth, and their ratio indicate intestinal integrity; longer villi enhance nutrient digestion and absorption, promoting animal growth [53]. Under DEX treatment, villus height and the villus height-to-crypt depth ratio in the duodenum and jejunum dropped significantly, and villus height in the ileum also trended downward. This suggests that DEX compromised intestinal morphology and hindered nutrient uptake. However, RES supplementation significantly restored villus height and the villus height-to-crypt depth ratio in the ileum, highlighting its capacity to protect intestinal structure—a finding consistent with previous reports showing that RES alleviates structural damage under heat stress [54], LPS challenges [20], and other stressors. This broad protective function is considered to involve antioxidant enzymes (GPX2, CAT, SOD), tight junction proteins (ZO-1, Occludin), and the nuclear receptor transcription factor PPAR-γ. ZO-1 and Occludin maintain the integrity of cell junctions, ensuring barrier function and preventing toxins and pathogens from entering the bloodstream [55]. In our experiments, DEX downregulated Occludin mRNA in the duodenum and appeared to lower ZO-1 mRNA in the ileum, indicating a compromised barrier. By contrast, RES supplementation upregulated Occludin mRNA in both the duodenum and ileum and showed a trend toward increasing ZO-1 mRNA in the ileum—consistent with our previous findings on RES-mediated barrier repair and its reported capacity to protect broiler intestines against LPS challenges [19]. Additionally, RES supplementation increased SOD mRNA in the duodenum as well as GPX2, SOD, and CAT mRNA in the ileum, suggesting an enhanced antioxidant defense under DEX induction. Such observations mirror those of other research, which shows that RES can mitigate oxidative stress in heat-stressed ducks [52] and reduce heat-induced intestinal damage in broilers [19]. PPAR-γ, known to reduce oxidative stress and inflammation while maintaining cellular homeostasis [56], was also upregulated by 0.1% RES in our study. Other reports confirm that RES can alleviate intestinal inflammation and barrier defects in colitis models, indicating its potential anti-inflammatory properties [57]. Overall, our findings demonstrate that under DEX-induced oxidative stress, RES alleviates oxidative damage in the broiler intestine, preserves the structural integrity of the small intestine, and offers additional benefits for intestinal immunity and inflammatory regulation.

Overall, this study has several limitations. First, for yellow-feathered broilers at 57 and 66 days of age, a factorial statistical framework with Treatment × Day would have been more appropriate than using only one-way ANOVA. Second, in poultry, UA is a more specific indicator of renal impairment than BUN; in the present study, only BUN was measured, which represents a limitation. Future work should address these shortcomings by incorporating a Treatment × Day design and including UA measurements to provide a more comprehensive assessment of renal function.

## 5. Conclusions

This study demonstrates that short-term supplementation with varying doses of RES significantly alleviates DEX-induced oxidative stress and intestinal morphological damage in yellow-feathered broilers. By improving antioxidant parameters, intestinal structure, and the expression of related genes, RES markedly increased average daily gain and enhanced meat quality. Different doses of RES exhibited varying degrees of mitigation, with the optimal supplementation level identified as 1000 mg/kg to 2000 mg/kg. These findings provide a novel strategy for short-term improvement of production performance and meat quality in broilers under oxidative stress, carrying substantial economic value.

## Figures and Tables

**Table 1:** Composition and nutrient levels of the basal diet.

Ingredients	Content (%)	Nutrient Levels	Content (%)
Corn	70.80	Metabolic energy (MJ/kg)	13.04
Soybean meal	16.90	crude protein	17.19
Corn gluten meal	6.00	calcium	0.75
Lard oil	2.50	Total phosphorus	0.49
Stone powder	1.31	Available phosphorus	0.28
CaHPO_4_	1.00	lysine	0.95
Lysine (65%)	0.60	sulfur-containing amino acid	0.75
Salt	0.27		
Dl-Methionine (98%)	0.18		
Baking soda	0.12		
Choline chloride (50%)	0.10		
Threonine	0.07		
Premix ^1^	0.15		
Total	100.00		

^1^ The premix provided the following per kg of diet: VA, 11,200 IU; VD3, 2800 IU; VE, 25 mg; VK3, 2 mg; VB1, 2 mg; VB2, 8 mg; VB6, 4 mg; VB12, 0.02 mg; D-pantothenic acid, 20 mg; niacin, 50 mg; folic acid, 1 mg; biotin, 0.2 mg; Fe, 60 mg; Cu, 10 mg; Mn, 80 mg; Zn, 60 mg; I, 0.2 mg; Se, 0.3 mg.

**Table 2:** Primer sequences, accession numbers, and sizes of the products ^1^.

Gene	Accession Number	Primer Sequences 5′→3′	Product Size (bp)
*GAPDH*	NM_204305.2	F: GGGCACGCCATCACTATCTT R: TCACAAACATGGGGGCATCA	187
*SOD*	NM_205064.2	F: CCCTTTGCAGTCACATTGCC R: CCACAAGCTAAACGAGGTCCA	198
*CAT*	NM_001031215.2	F: ATTCCTGAAAGAGTTGTGCAT R: TGTTCAAACACTTTCGCCTTG	101
*GPX2*	NM_001277854.3	F: CAACCAGCTGCAGGCACGCTA R: ATCTCCTCGTTGGTGCCGTT	96
*ZO-1*	XM_040706827.2	F: CTTCAGGTGTTTCTCTTCCTCCTC R: CTGTGGTTTCATGGCTGGATC	131
*PPAR-γ*	NM_001001460.2	F: CTCAGACAAATTGTAACGGAA R: GATAAGAACTACTATCGCCAT	131
*Occludin*	NM_205128.1	F: TTCGTCATGCTCATCGCCTC R: TCCACGGTGCAGTAGTGGTA	158

^1^ The primers were designed and synthesized by Shanghai Shenggong Biological Engineering Co., Ltd. (Shanghai, China). F, forward; R, reverse; *GAPDH*, Glyceraldehyde-3-Phosphate Dehydrogenase; *SOD*, Superoxide Dismutase; *CAT*, Catalase; *GPX2*, Glutathione Peroxidase 2; *ZO-1*, Zona Occludens 1; *PPAR-γ*, Peroxisome Proliferator-Activated Receptor Gamma.

**Table 3:** Effects of dietary resveratrol supplementation on the growth performance of dexamethasone-challenged yellow-feathered broilers.

Items	CON	DEX	DR1	DR2	DR3	SEM ^1^	*p* Value ^2^		
							ANOVA	Linear	Quadratic
Days 1–5									
BW1 (g)	1527.38	1488.38	1558.88	1554.00	1564.13	26.41	0.90	0.44	0.64
BW5 (g)	1687.38	1411.13	1573.38	1574.88	1571.00	33.33	0.13	0.16	0.28
ADG_1–5_ (g/d)	32.00 ^a^	−15.45 ^c^	2.90 ^b^	4.18 ^b^	1.38 ^b^	3.52	<0.01	0.07	0.10
ADFI_1–5_ (g/d)	135.30	150.33	155.33	149.01	157.42	3.09	0.43	0.57	0.77
FCR_1–5_	4.06	102.12	2.93	30.33	−1.99	19.61	0.42	0.19	0.49
Mortality (%)	0	4.17	0	4.17	4.17				
Days 6–14									
BW6 (g)	1687.66	1410.20	1573.66	1574.11	1570.86	32.86	0.13	0.16	0.28
BW14 (g)	2140.00 ^a^	1770.50 ^b^	1940.25 ^b^	1926.90 ^b^	1887.13 ^b^	36.76	0.02	0.34	0.19
ADG_6–14_ (g/d)	50.29	39.93	40.76	39.11	35.13	1.92	0.14	0.41	0.58
ADFI_6–14_ (g/d)	171.37 ^a^	152.13 ^b^	150.40 ^b^	147.02 ^b^	140.96 ^b^	3.00	0.01	0.17	0.71
FCR_6–14_	3.51	4.25	3.97	4.12	4.34	0.20	0.73	0.85	0.62
Mortality (%)	0	2.08	0	0	0				
Days 1–14									
ADG_1–14_ (g/d)	43.76 ^a^	20.15 ^c^	27.24 ^b^	26.64 ^b^	23.07 ^bc^	1.55	<0.01	0.36	0.01
ADFI_1–14_ (g/d)	158.49 ^a^	151.48 ^ab^	152.16 ^ab^	147.73 ^ab^	146.84 ^b^	1.37	0.05	0.17	0.79
FCR_1–14_	3.68 ^c^	7.95 ^a^	5.74 ^b^	5.84 ^b^	6.75 ^ab^	0.32	<0.01	0.20	0.01
Mortality (%)	0	6.25	0	4.17	4.17				

^1^ Standard error means, same below. ^2^ ANOVA, *p* value for the overall treatment effect from a one-way analysis of variance; Linear, *p* value for the linear orthogonal polynomial contrast across dietary RES doses; Quadratic, *p* value for the quadratic orthogonal polynomial contrast across dietary RES doses. Same below. ^abc^ Means in the same row with different superscripts differ (*p* < 0.05, *n* = 8). Same below. BW1, body weight on day 1; BW5, body weight on day 5 (pre-sampling); BW6, body weight on day 6 (post-sampling); BW14, body weight on day 14; ADG_1–5_, average daily gain during days 1–5; ADFI_1–5_, average daily feed intake during days 1–5; FCR_1–5_, feed conversion ratio during days 1–5; ADG_6–14_, average daily gain during days 6–14; ADFI_6–14_, average daily feed intake during days 6–14; FCR_6–14_, feed conversion ratio during days 6–14; ADG_1–14_, average daily gain during days 1–14; ADFI_1–14_, average daily feed intake during days 1–14; FCR_1–14_, feed conversion ratio during days 1–14.

**Table 4:** Effects of dietary resveratrol supplementation on the plasma biochemical parameters of dexamethasone-challenged yellow-feathered broilers.

Items	CON	DEX	DR1	DR2	DR3	SEM	*p* Value		
							ANOVA	Linear	Quadratic
Day 6 ^1^									
CS (ng/L)	0.27 ^d^	0.58 ^a^	0.43 ^c^	0.45 ^bc^	0.51 ^b^	0.01	<0.01	<0.01	<0.01
Day 14									
CS (ng/L)	0.30 ^c^	0.55 ^a^	0.46 ^b^	0.51 ^ab^	0.46 ^b^	0.01	<0.01	<0.01	0.11
TP (g/L)	38.14 ^ab^	36.39 ^b^	39.78 ^ab^	40.76 ^a^	41.46 ^a^	0.99	<0.01	<0.01	0.20
ALB (g/L)	17.68 ^ab^	16.65 ^b^	18.93 ^a^	18.56 ^a^	17.90 ^ab^	0.44	0.01	0.13	<0.01
GLB (g/L)	20.46	19.74	20.85	22.20	23.56	0.95	0.06	<0.01	0.90
A/G	0.88	0.85	0.94	0.85	0.77	0.046	0.21	0.16	0.10
Cr (μmol/L)	2.38	3.12	1.88	2.50	4.00	0.53	0.10	0.24	0.03
BUN (mmol/L)	0.50 ^b^	0.68 ^a^	0.51 ^b^	0.41 ^b^	0.44 ^b^	0.03	<0.01	<0.01	<0.01
Glu (mmol/L)	10.19	10.86	10.25	10.49	10.32	0.29	0.51	0.29	0.44

^1^ *n* = 4 for CS on day 6; *n* = 8 for all other measurements. CS, corticosterone; TP, total protein; ALB, albumin; GLB, globulin; A/G, albumin-to-globulin ratio; Cr, creatinine; BUN, blood urea nitrogen; Glu, glucose. ^abcd^ Means in the same row with different superscripts differ.

**Table 5:** Effects of dietary resveratrol supplementation on the antioxidant capacity of serum, liver, and thigh muscle in dexamethasone-challenged yellow-feathered broilers.

Items	CON	DEX	DR1	DR2	DR3	SEM	*p* Value		
							ANOVA	Linear	Quadratic
Serum									
MDA (ng/L)	2.26	3.92	2.30	2.92	2.88	0.57	0.31	0.41	0.24
SOD (U/mL)	183.52 ^a^	165.84 ^b^	172.00 ^b^	179.29 ^a^	172.83 ^b^	2.44	<0.01	0.01	0.01
CAT (U/mL)	9.49	8.85	12.90	13.23	12.67	2.13	0.46	0.21	0.27
GSH-Px (U/mL)	1903.28 ^b^	1664.75 ^b^	2883.20 ^a^	2150.41 ^b^	2152.87 ^b^	116.61	<0.01	0.26	<0.01
Liver									
MDA (ng/L)	2.59 ^b^	4.94 ^a^	3.42 ^ab^	3.77 ^ab^	4.12 ^ab^	0.38	0.01	0.35	0.07
SOD (U/mL)	36.08 ^c^	33.94 ^c^	48.31 ^a^	40.40 ^b^	43.04 ^b^	1.19	<0.01	<0.01	<0.01
CAT (U/mL)	12.69 ^a^	8.43 ^b^	12.56 ^a^	11.79 ^a^	10.61 ^ab^	0.56	<0.01	0.09	0.10
GSH-Px (U/mL)	61.71 ^b^	51.58 ^b^	73.76 ^a^	64.38 ^ab^	66.94 ^ab^	3.89	<0.01	0.06	0.03
Thigh muscle									
MDA (ng/L)	5.28 ^b^	8.26 ^a^	8.03 ^a^	5.53 ^ab^	5.08 ^b^	0.66	<0.01	<0.01	0.87
SOD (U/mL)	73.37 ^a^	58.95 ^b^	83.53 ^a^	84.30 ^a^	79.82 ^a^	2.75	<0.01	<0.01	<0.01
CAT (U/mL)	25.87 ^a^	16.88 ^b^	26.45 ^a^	29.66 ^a^	25.46 ^a^	1.86	<0.01	<0.01	<0.01
GSH-Px (U/mL)	17.10 ^a^	8.24 ^b^	18.46 ^a^	16.18 ^a^	15.18 ^a^	1.19	<0.01	<0.01	<0.01

MDA, malondialdehyde; SOD, superoxide dismutase; CAT, catalase; GSH-Px, glutathione peroxidase. ^abc^ Means in the same row with different superscripts differ.

**Table 6:** Effects of dietary resveratrol supplementation on the intestinal antioxidant capacity of dexamethasone-challenged yellow-feathered broilers ^1^.

Items	CON	DEX	DR1	DR2	DR3	SEM	*p* Value		
							ANOVA	Linear	Quadratic
Duodenum									
MDA (nmol/mg prot)	0.12 ^b^	0.51 ^a^	0.27 ^ab^	0.27 ^ab^	0.31 ^ab^	0.04	<0.01	0.08	0.06
SOD (U/mg prot)	17.37 ^b^	17.07 ^ab^	18.17 ^ab^	19.30 ^ab^	19.58 ^a^	0.50	0.02	0.01	0.51
Jejunum									
MDA (nmol/mg prot)	0.24 ^b^	0.41 ^a^	0.23 ^b^	0.22 ^b^	0.20 ^b^	0.03	<0.01	<0.01	<0.01
SOD (U/mg prot)	17.37	19.93	18.22	18.86	20.11	0.73	0.20	0.80	0.16
Ileum									
MDA (nmol/mg prot)	0.13 ^b^	0.52 ^a^	0.20 ^b^	0.39 ^ab^	0.29 ^ab^	0.05	<0.01	0.14	0.13
SOD (U/mg prot)	18.21	17.92	18.35	19.94	19.12	0.47	0.14	0.09	0.35

^1^ *n* = 4. MDA, malondialdehyde; SOD, superoxide dismutase. ^ab^ Means in the same row with different superscripts differ.

**Table 7:** Effects of dietary resveratrol supplementation on the meat quality of dexamethasone-challenged yellow-feathered broilers.

Items	CON	DEX	DR1	DR2	DR3	SEM	*p* Value		
							ANOVA	Linear	Quadratic
Thigh muscle									
a*	8.08 ^a^	5.98 ^b^	7.33 ^ab^	7.45 ^ab^	7.60 ^a^	0.35	<0.01	<0.01	0.14
b*	11.31 ^a^	7.56 ^c^	9.35 ^b^	8.73 ^bc^	9.59 ^b^	0.30	<0.01	<0.01	0.19
L*	54.75 ^ab^	53.37 ^b^	54.64 ^ab^	55.09 ^a^	54.01 ^ab^	0.33	0.01	0.15	<0.01
pH_45min_	6.53 ^b^	6.79 ^a^	6.42 ^b^	6.52 ^b^	6.47 ^b^	0.06	<0.01	<0.01	0.02
pH_24h_	5.89 ^c^	5.99 ^ab^	5.94 ^bc^	6.02 ^a^	5.94 ^bc^	0.02	<0.01	0.42	0.36
Drip loss (%)	2.62 ^ab^	3.02 ^a^	2.16 ^b^	2.26 ^b^	2.14 ^b^	0.17	<0.01	<0.01	0.05
Shear force (N)	19.77	19.10	20.88	20.79	20.31	1.35	0.89	0.51	0.35
Pectoralis									
a*	10.69 ^a^	8.88 ^c^	10.47 ^ab^	8.78 ^c^	9.16 ^bc^	0.34	<0.01	0.58	0.10
b*	10.26 ^a^	8.07 ^b^	9.47 ^ab^	9.17 ^ab^	10.08 ^a^	0.37	<0.01	<0.01	0.57
L*	53.66 ^a^	51.39 ^b^	54.31 ^a^	54.73 ^a^	54.88 ^a^	0.49	<0.01	<0.01	0.01
pH_45min_	6.41 ^ab^	6.43 ^a^	6.32 ^bc^	6.35 ^ac^	6.28 ^c^	0.02	<0.01	<0.01	0.33
pH_24h_	6.23	6.28	6.29	6.23	6.20	0.03	0.23	0.02	0.35
Drip loss (%)	1.43	1.65	1.66	1.74	1.64	0.13	0.56	0.90	0.71
Shear force (N)	18.91	22.31	19.75	21.13	18.64	0.99	0.07	0.04	0.98

a*, redness; b*, yellowness; L*, lightness; pH_45min_, pH at 45 min postmortem; pH_24h_, pH at 24 h postmortem. ^abc^ Means in the same row with different superscripts differ.

## Data Availability

The original contributions presented in this study are included in the article. Further inquiries can be directed to the corresponding author.
